# Cerebrospinal fluid levels of neurogranin and YKL-40 in mild cognitive impairment due to Alzheimer's disease or vascular dementia

**DOI:** 10.1177/13872877251411336

**Published:** 2026-01-13

**Authors:** Ulla Andin, Svante Lifvergren, Henrik Zetterberg, Kaj Blennow, Robert Lundin, Johan Svensson

**Affiliations:** 1 Department of Internal Medicine, Skaraborg Central Hospital, Lidköping, Sweden; 2 Collaborative Platform for Healthcare Improvement (CHI), Chalmers University of Technology, Gothenburg, Sweden; 3 Institute of Neuroscience and Physiology, Department of Psychiatry and Neurochemistry, Sahlgrenska Academy, University of Gothenburg, Mölndal, Sweden; 4 Clinical Neurochemistry Laboratory, Sahlgrenska University Hospital, Mölndal, Sweden; 5 Department of Neurodegenerative Disease, UCL Institute of Neurology, Queen Square, London, UK; 6 UK Dementia Research Institute at UCL, London, UK; 7 Hong Kong Center for Neurodegenerative Diseases, InnoHK, Hong Kong, China; 8 Wisconsin Alzheimer's Disease Research Center, University of Wisconsin School of Medicine and Public Health, University of Wisconsin-Madison, Madison, WI, USA; 9 Department of Psychiatry, Skaraborg Central Hospital, Skövde, Sweden; 10 Department of Internal Medicine and Clinical Nutrition, Institute of Medicine, Sahlgrenska Academy, University of Gothenburg, Gothenburg, Sweden; 11 Department of Internal Medicine, Skaraborg Central Hospital, Skövde, Sweden

**Keywords:** Alzheimer's disease, cerebrospinal fluid, mild cognitive impairment, neurogranin, stable mild cognitive impairment, vascular dementia, YKL-40

## Abstract

**Background:**

Markers of synaptic degeneration and neuroinflammation have been investigated in memory clinic cohorts, but less is known about their role in community-dwelling subjects.

**Objective:**

To investigate baseline cerebrospinal fluid (CSF) levels of neurogranin and YKL-40 in community-dwelling subjects with mild cognitive impairment (MCI) who had not yet sought help for their cognitive decline.

**Methods:**

We recruited and characterized 107 subjects, who at the clinical baseline examination were found to have MCI. Based on the clinical progression at a 3-year follow-up, the individuals were classified as MCI-Alzheimer's disease (MCI-AD, n = 40), MCI-vascular dementia (MCI-VaD, n = 25), and stable MCI (sMCI, n = 42).

**Results:**

Baseline CSF neurogranin level was elevated in the MCI-AD group compared with the MCI-VaD and sMCI groups (p = 0.02 and p < 0.001, respectively), and baseline CSF YKL-40 level was higher in the MCI-AD group than in the sMCI group (p = 0.01). Neurogranin, and to a lesser extent YKL-40, correlated positively with CSF levels of total tau and phosphorylated tau_181_ in all study groups. However, in receiver operator characteristics analyses, neurogranin and YKL-40 used alone or in combination had a moderate diagnostic accuracy that was lower than that of the core AD biomarkers (amyloid-β_42_, total tau, and phosphorylated tau_181_).

**Conclusions:**

This study shows that neurogranin and YKL-40 in CSF are elevated in MCI-AD compared with sMCI, and neurogranin was also higher in MCI-AD than in MCI-VaD. Neurogranin and YKL-40 had a moderate diagnostic accuracy, but they could still be of value to characterize the clinical consequences of postsynaptic dysfunction and neuroinflammation.

## Introduction

Alzheimer's disease (AD) followed by vascular dementia (VaD) are leading causes of cognitive impairment in older individuals.^1–3^ In the AD brain, there are accumulation of amyloid-β (Aβ), neurofibrillary tangles of hyperphosphorylated tau, neurodegeneration, and neuroinflammation.^1,2^ Although cerebrospinal fluid (CSF) levels of the core AD biomarkers [Aβ_42_, total-tau (t-tau) and phosphorylated tau protein_181_ (p-tau_181_)] have a high ability to separate AD from healthy individuals,^1,2^ they provide limited information of other aspects of AD development such as synaptic dysfunction and neuroinflammation.^4^

Neurogranin is a postsynaptic protein concentrated in dendritic spines, where it is involved in postsynaptic signaling pathways by regulating the calcium-binding protein calmodulin.^5^ Studies based on memory clinic cohorts have shown that CSF neurogranin levels are higher in AD dementia compared with healthy controls^6–14^ or compared with individuals under evaluation for other neurological disorders.^6,7,13,15,16^ The increase in CSF neurogranin has been specific for AD compared with other neurodegenerative conditions, possibly with the exception of Creutzfeldt-Jakob disease.^17^ However, CSF neurogranin levels are gradually increased during the progression of AD neuropathology. In a study using Aβ positron emission tomography (PET), CSF neurogranin, as a function of Aβ load, gradually increased first when brain Aβ load was higher than the threshold for Aβ positivity.^18^ Furthermore, using the amyloid plaque (A)/tau-tangle (T)/neurodegeneration (N) (ATN) system, CSF neurogranin was lowest in the (A−/T−/N−) and (A+/T−/N−) groups and then increased with the appearance of T+ and N+.^19,20^ Finally, although the results have not been fully consistent,^21^ CSF neurogranin has been higher in AD dementia than in manifest VaD.^22–24^

AD neuropathology induces immune responses in the brain, which include activation of glial cells like microglia and astrocytes.^25^ The glycoprotein YKL-40, or chitinase-3-like protein 1, is upregulated in inflammatory conditions.^26^ In the brain, YKL-40 expression is abundant in astrocytes near Aβ plaques.^27^ Therefore, YKL-40 can be considered as a marker of neuroinflammation.^27^ CSF YKL-40 level is elevated in AD dementia compared with controls,^23,^^28–33^ and is higher in prodromal AD or AD dementia compared with preclinical AD.^34,35^ Furthermore, higher CSF YKL-40 has also been found in frontotemporal dementia (FTD).^33,36^ Relatively few studies have investigated CSF YKL-40 levels in VaD patients and with somewhat diverging results.^23,27,^^37–39^

In summary, little is known about CSF levels of neurogranin and YKL-40 in subjects with mild cognitive impairment (MCI) who have not sought health care. We therefore recruited community-dwelling individuals (n = 107) who had not yet sought help for their cognitive decline. Our major aim was to determine whether CSF levels of neurogranin or YKL-40 could distinguish MCI-AD (n = 40) from MCI-VaD (n = 25) and stable MCI (sMCI, n = 42). We hypothesized that an increase in CSF neurogranin level, but not in CSF YKL-40 level, would be specific for MCI-AD.

## Methods

### Patients

The study included 107 community-dwelling individuals (47 men and 60 women) who were recruited by advertisements in local newspapers and at primary care units. None of the subjects had a previous diagnosis of a specific cognitive disorder and none of the participants were under evaluation for cognitive decline. The inclusion criteria were age ≥ 60 years, cognitive impairment [Mini-Mental State Examination (MMSE) ≥ 24], living at home, and ability to give oral and written informed consent. Exclusion criteria were severe somatic or psychiatric disease, drug abuse, blindness, deafness, and specific cognitive disorders other than AD and VaD. In the present study, we also excluded individuals with clinically manifest AD dementia or VaD dementia. The included patients were evaluated at baseline at Skaraborg Central Hospital, Falköping, Sweden by specialized physicians. In addition, a clinical follow-up visit was performed after 3 years to evaluate if the participants had progressed to dementia. After these procedures were completed, the included individuals were diagnosed with MCI-AD (n = 40), MCI-VaD (n = 25), and sMCI (n = 42). Furthermore, in the MCI-AD group (n = 40), 26 of the individuals had MCI-AD without major concomitant cerebral vascular pathology and 14 subjects suffered from MCI-mixed dementia (combined MCI-AD and MCI-VaD).

MCI was diagnosed according to the Peterson criteria.^40^ The presence or absence of dementia was diagnosed according to the Diagnostic and Statistical Manual of Mental Disorders, Fifth Edition (DSM-V). The specially trained physician who set the specific diagnoses had access to CSF Aβ_42_/Aβ_40_ ratio, but was otherwise blinded to CSF biomarker data. AD was diagnosed using the NINCDS-ADRDA criteria.^41^ Moreover, for an AD diagnosis, it was required that the patient had no or mild amount of white matter hyperintensities (WMHs) according to the Fazekas scale^42^ on neuroimaging, and predominant parietotemporal lobe symptoms. In addition, amyloid positivity (CSF Aβ_42_/Aβ_40_ ratio ≤ 0.072) was required for an AD diagnosis. Mixed dementia was diagnosed if AD patients also fulfilled the criteria of VaD (see below). In the present study, the 14 participants with MCI-mixed dementia (combined MCI-AD and MCI-VaD) were included in the MCI-AD group.

The diagnosis of VaD included cortical VaD (cVaD) classified according to the NINDS-AIREN criteria^43^ and subcortical vascular dementia (SVD) as defined by Erkinjuntti et al.^44^ Furthermore, our classification of SVD is in line with the Vascular Impairment of Cognition Classification Consensus Study (VICCCS), in which SVD, denominated subcortical ischemic vascular dementia, was one of the entities.^45^ More specifically: for SVD, the patient must have moderate or severe amount of WMHs according to Fazekas classification^42^ and predominant frontal lobe symptoms. A diagnosis of cVaD was set if the dementia onset was stroke-related (single- or multi-infarct). MCI patients who had not converted to dementia at the 3-year follow-up and did not fulfill the criteria of AD or VaD were defined as sMCI.

We originally evaluated 126 individuals. However, we excluded subjects who at baseline suffered from clinically manifest AD dementia (n = 7) and clinically manifest VaD dementia (n = 4) as well as subjects with specific cognitive disorders other than AD and VaD [primary progressive aphasia (PPA, n = 2), Lewy body dementia (LBD, n = 1), and unknown type of dementia (n = 1)]. Finally, three individuals with clinically diagnosed MCI-AD were excluded due to a CSF Aβ_42_/Aβ_40_ ratio > 0.072 and one subject with MCI-VaD was excluded based on a CSF Aβ_42_/Aβ_40_ ratio < 0.072. Therefore, the study population ultimately consisted of 107 individuals with MCI. The MCI-AD group (n = 40) comprised MCI-AD without any major cerebral vascular comorbidity (n = 26) and MCI-mixed dementia (combined MCI-AD and MCI-VaD, n = 14). The MCI-VaD group (n = 25) consisted of 18 subjects with MCI-SVD and 7 individuals with MCI-cVaD. The sMCI group comprised 42 subjects.

## Ethical considerations

The study was approved by the ethical committee of University of Gothenburg (1177–16; 19 March 2017) and the Swedish Ethical Review Authority (2020–01445; 29 June 2020). Oral and written informed consent was obtained from all participants. The study was conducted according to the Declaration of Helsinki. Furthermore, all study procedures were performed in adherence to ethical standards and the ethical approvals for the present study.

### Physical examination and neuropsychological tests

At baseline, the patients were evaluated by physical examination, neuropsychological tests, and lumbar puncture. Body mass index (BMI) was calculated as the weight in kilograms divided by the height in meters squared.

MMSE was performed to estimate global cognitive function,^46^ Montreal Cognitive Assessment (MoCA) to evaluate visuospatial construction and executive functions,^47^ and Trail Making Test A (TMT-A) to assess visuoperceptual speed.^48^ In addition, the Cognitive Assessment Battery (CAB) was used.^49^ From the CAB, we used immediate recall, delayed recall, clock drawing (clox) and cube (reflecting visuospatial functions), symbol digit modalities test (SDMT; speed and attention), Token test (language comprehension), naming (language functions) and Stroop III (executive functions).

### CSF sampling

At baseline, in the morning after an overnight fast, lumbar puncture was performed in the L3/L4 or L4/L5 interspace. The first 12 mL of CSF was collected in polypropylene tubes and immediately transported to the laboratory for centrifugation at 2.000 g at +4 °C for 10 min. The supernatant was pipetted off, gently mixed to avoid possible gradient effects, and aliquoted in polypropylene tubes that were stored at −80 °C pending biochemical analyses, without being thawed and re-frozen.

### Biochemical procedures

All biochemical analyses were conducted by experienced laboratory technicians blinded to clinical information. CSF concentrations of Aβ_42_, Aβ_42_/Aβ_40_ ratio, t-tau, and p-tau_181_ were determined using electro-chemiluminescence immunoassays (ECLIA) on a Lumipulse G600 II instrument, as previously described.^50^ Neurofilament light chain (NFL) and neurogranin concentrations in CSF were measured using in house-developed enzyme-linked immunosorbent assays (ELISA), as previously described.^51,52^ CSF YKL-40 concentration was measured using a commercial ELISA (R&D systems, Minneapolis, MN). All measurements were performed in one round of experiments using one batch of reagents. Intra-assay coefficients of variation were <10%. Calibrators and quality control (QC) samples were run in duplicates and samples were run in singlicates.

### Statistical analyses

Statistical evaluation was performed using SPSS version 29 (IBM Corp., Armonk, NY). The descriptive statistical results are given as the median (25th-75th percentiles) if not otherwise stated. In the present study, we had complete data (n = 107) in terms of all CSF biomarker variables (neurogranin, YKL-40, and the core AD biomarkers). Between-group differences for continuous variables were assessed using the Kruskal Wallis test for multiple variables followed by the Mann-Whitney U test for pair-wise comparisons. For categorical variables, differences between groups were evaluated using chi-square tests. Correlations were sought using the Spearman rank order correlation test. The relationships between sensitivity and specificity between study groups were analyzed using receiver operator characteristics (ROC) analysis. In these analyses, we calculated area under the receiver operating characteristics curve (AUROC) and 95% confidence intervals (CIs). Significance was obtained if the two-tailed p-value was ≤ 0.05.

## Results

### Baseline characteristics and neuropsychological test results

Participants with MCI-AD and MCI-VaD were older than subjects with sMCI (both p < 0.01; Table 1). The groups were comparable at baseline in terms of gender, education, BMI, and scores of MMSE, MoCA, and TMT-A (Table 1). For the tests included in the CAB, the MCI-VaD group had a lower (worse) SDMT score compared with the sMCI group (p = 0.01). Immediate recall, delayed recall, clox and cube, Token test, naming and Stroop III did not differ between groups at baseline (Table 1).

**Table 1.** Baseline clinical characteristics including neuropsychological test results in 107 subjects with MCI who had not yet sought help for their cognitive decline.

	MCI-AD (n = 40)	MCI-VaD (n = 25)	sMCI (n = 42)	p
Age (y)	76 (70–79)^a^	76 (72–81)^a^	72 (68–75)	<0.01
Men/women, n (%)	19 (48%)/21 (52%)	7 (28%)/18 (72%)	21 (50%)/21 (50%)	0.18
Education (years)	12 (9–15)	9 (9–14)	12 (9–15)	0.14
BMI (kg/m^2^)	24.9 (23.2–27.8)	27.2 (23.8–29.9)	25.9 (23.9–30.4)	0.12
*Neuropsychological tests*				
MMSE	29 (28–30)	29 (28–29)	29 (28–29)	0.57
MoCA	28 (25–29)	27 (25–28)	29 (27–29)	0.16
TMT-A	43 (34–53)	45 (39–56)	37 (31–47)	0.07
Cognitive Assessment Battery				
SDMT	37 (30–41)	32 (29–38)^b^	39 (32–44)	0.03
Immediate recall	8 (6–10)	8 (6–10)	8 (5–10)	0.86
Delayed recall	12 (9–15)	14 (8–17)	11 (7–14)	0.40
Clox and cube	12 (12–12)	12 (12–12)	12 (12–12)	0.53
Token test	6 (5–6)	5 (4–6)	6 (5–6)	0.09
Naming	29 (28–30)	28 (26–29)	29 (27–29)	0.10
Stroop III	33 (25–44)	36 (28–46)	30 (26–37)	0.19

Values are given as the median (25th-75th percentiles). Between-group differences were assessed using the Kruskal-Wallis test for multiple variables, followed by the Mann-Whitney U test for pair-wise comparisons.

^a^ p < 0.01 vs. sMCI,

^b^ p = 0.01 vs. sMCI.

AD: Alzheimer's disease; BMI: body mass index; MCI: mild cognitive impairment; MMSE: Mini-Mental State Examination; MoCA: Montreal Cognitive Assessment; SDMT: Symbol Digit Modalities Test; sMCI: stable MCI; TMT-A: Trail Making Test A; VaD: vascular dementia.

### CSF biomarker levels at baseline

**Figure 1.**
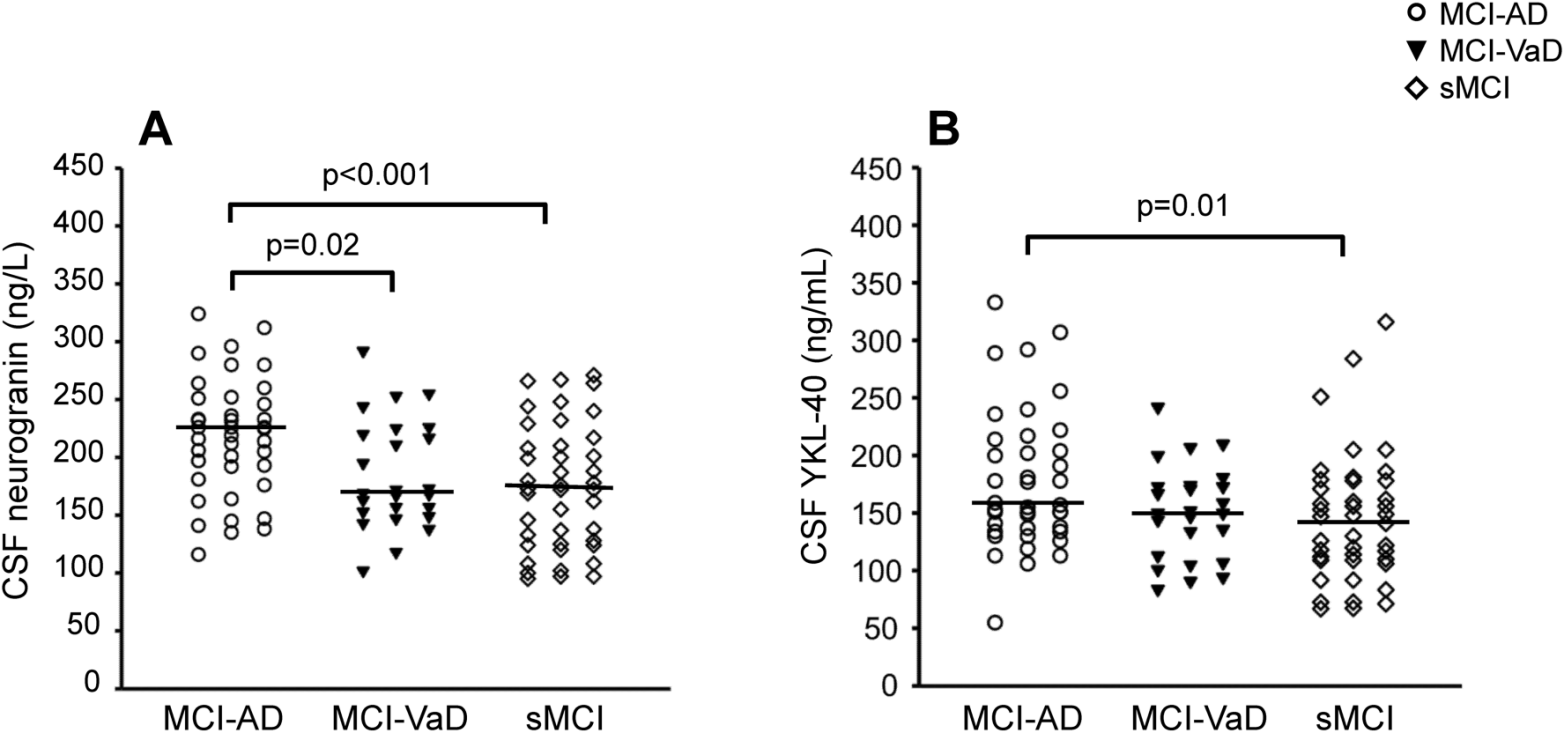
Cerebrospinal fluid (CSF) levels of neurogranin and YKL-40 were elevated in mild cognitive impairment (MCI) due to Alzheimer's disease (AD) compared with stable MCI (sMCI), and neurogranin was also higher in MCI-AD than in MCI-vascular dementia (MCI-VaD). (A) CSF neurogranin concentration and (B) CSF YKL-40 concentration in MCI-AD (n = 40), MCI-VaD (n = 25), and sMCI (n = 42). The horizontal lines represent the median values. Between-group differences were assessed using the Kruskal-Wallis test for multiple variables, followed by the Mann-Whitney U test for pair-wise comparisons.

CSF neurogranin level was higher in the MCI-AD group than in the MCI-VaD and sMCI groups (p = 0.02 and p < 0.001, respectively; Figure 1A and Table 2). CSF YKL-40 level was higher in the MCI-AD group than in the sMCI group (p = 0.01) (Figure 1B and Table 2). In terms of the core CSF AD biomarkers, the AD group had lower levels of Aβ_42_/Aβ_40_ ratio and Aβ_42_ in CSF and higher CSF t-tau and p-tau_181_ compared with the other study groups (all p < 0.001; Table 2). In addition, CSF Aβ_42_ level was higher in the MCI-VaD group than in the sMCI group (p = 0.04). CSF NFL level was higher in the MCI-AD and MCI-VaD groups compared with the sMCI group (p < 0.001 and p < 0.01, respectively; Table 2).

**Table 2.** CSF biomarkers at baseline in 107 subjects with MCI who had not yet sought help for their cognitive decline.

	MCI-AD (n = 40)	MCI-VaD (n = 25)	sMCI (n = 42)	p
Neurogranin (ng/L)	222 (184–250)^a^^,^^b^	169 (151–223)	173 (125–212)	<0.01
YKL-40 (ng/mL)	156 (135–212)^c^	150 (110–174)	144 (109–178)	0.03
Aβ_42_/Aβ_40_ ratio	0.051 (0.044–0.057)^b^^,^^d^	0.097 (0.091–0.110)	0.100 (0.094–0.110)	<0.001
Aβ_42_ (ng/L)	580 (460–648)^b^^,^^d^	1060 (900–1175)^e^	880 (790–1045)	<0.001
t-tau (ng/L)	385 (330–488)^b^^,^^d^	270 (225–320)	215 (160–310)	<0.001
p-tau_181_ (ng/L)	60 (50–72)^b^^,^^d^	46 (39–53)	41 (32–51)	<0.001
NFL (ng/L)	945 (715–1270)^b^	950 (720–1185)^c^	675 (408–890)	<0.001

Values are given as the median (25th-75th percentiles). Between-group differences were assessed using the Kruskal-Wallis test for multiple variables, followed by the Mann-Whitney U test for pair-wise comparisons.

^a^ p = 0.02 vs. MCI-VaD;

^b^ p < 0.001 vs. sMCI;

^c^ p ≤ 0.01 vs. sMCI;

^d^ p < 0.001 vs. MCI-VaD;

^e^ p = 0.04 vs. sMCI.

Aβ: amyloid-β; AD: Alzheimer's disease; CSF: cerebrospinal fluid; MCI: mild cognitive impairment; NFL: neurofilament light chain; P-tau_181_: phosphorylated tau_181_; sMCI: stable MCI; T-tau: total tau; VaD: vascular dementia.

We did not include CSF Aβ_42_/Aβ_40_ ratio in further analyses as an Aβ_42_/Aβ_40_ ratio ≤ 0.072 was required for a diagnosis of MCI-AD in the present study.

### Correlation analysis

**Figure 2.**
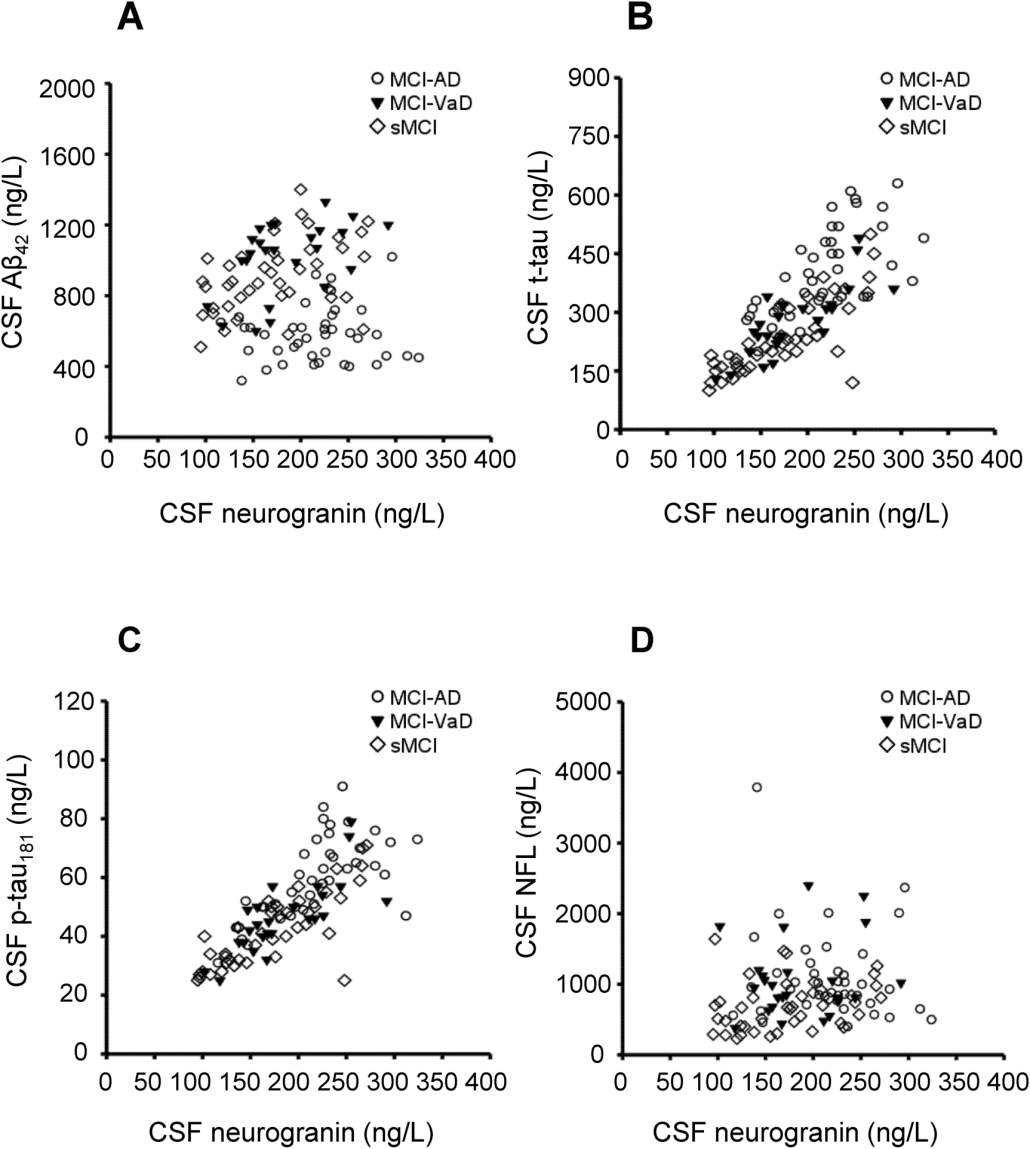
The correlations between CSF neurogranin concentrations and CSF concentrations of the biomarkers Aβ_42_, t-tau, p-tau_181_, and NFL. (A) CSF neurogranin was not correlated with CSF Aβ_42_ in the total study population (n = 107; r_s_ = −0.03) or in the MCI-AD group (n = 40, r_s_ = 0.00), whereas CSF neurogranin was positively correlated with CSF Aβ_42_ in the MCI-VaD group (n = 25; r_s_ = 0.51, p = 0.01) and in the sMCI group (n = 42; r_s_ = 0.43, p < 0.01). (B) The positive correlations between CSF neurogranin and CSF t-tau in the total study population (r_s_ = 0.80, p < 0.001), MCI-AD (r_s_ = 0.70, p < 0.001), MCI-VaD (r_s_ = 0.80, p < 0.001), and sMCI (r_s_ = 0.78, p < 0.001). (C) The positive correlations between CSF neurogranin and CSF p-tau_181_ in the total study population (r_s_ = 0.81, p < 0.001), MCI-AD (r_s_ = 0.71, p < 0.001), MCI-VaD (r_s_ = 0.80, p < 0.001), and sMCI (r_s_ = 0.77, p < 0.001). (D) CSF neurogranin was positively correlated with CSF NFL in the entire study population (r_s_ = 0.21, p = 0.03) and in the sMCI group (r_s_ = 0.36, p = 0.02), whereas CSF neurogranin was not correlated with CSF NFL in the MCI-AD group (r_s_ = −0.18) or in the MCI-VaD group (r_s_ = 0.09). Correlations were sought using the Spearman rank order correlation test. Aβ: amyloid-β; AD: Alzheimer's disease; CSF: cerebrospinal fluid; MCI: mild cognitive impairment; NFL: neurofilament light chain; P-tau_181_: phosphorylated tau_181_; sMCI: stable MCI; T-tau: total tau; VaD: vascular dementia.

**Figure 3.**
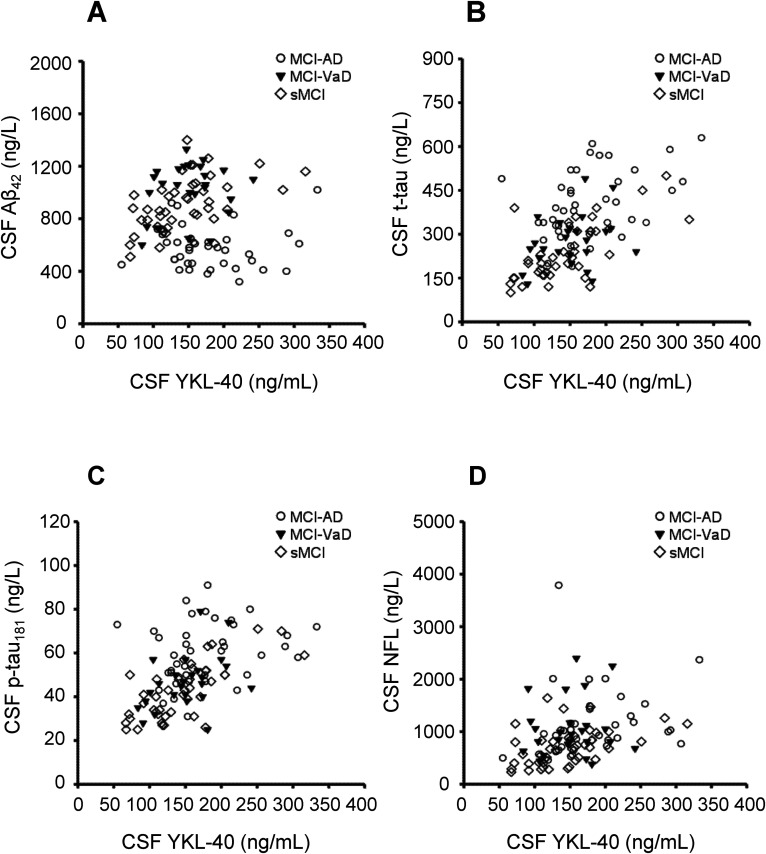
The correlations between CSF YKL-40 concentrations and CSF concentrations of the biomarkers Aβ_42_, t-tau, p-tau_181_, and NFL. (A) CSF YKL-40 was not correlated with CSF Aβ_42_ in the total study population (n = 107; r_s_ = −0.01), MCI-AD (n = 40, r_s_ = −0.20), or MCI-VaD (n = 25, r_s_ = 0.08), whereas CSF YKL-40 was positively correlated with CSF Aβ_42_ in the sMCI group (n = 42; r_s_ = 0.53, p < 0.001). (B) CSF YKL-40 was positively correlated with CSF t-tau in the total study population (r_s_ = 0.52, p < 0.001), MCI-AD (r_s_ = 0.44, p < 0.01), and sMCI (r_s_ = 0.64, p < 0.001), whereas YKL-40 did not correlate with t-tau in the MCI-VaD group (r_s_ = 0.24). (C) CSF YKL-40 was positively correlated with CSF p-tau_181_ in the total study population (r_s_ = 0.55, p < 0.001), MCI-VaD (r_s_ = 0.40, p < 0.05), and sMCI (r_s_ = 0.67, p < 0.001), whereas there was no significant correlation between YKL-40 and p-tau_181_ in the MCI-AD group (r_s_ = 0.30). (D) CSF YKL-40 was positively correlated with CSF NFL in the entire study population (r_s_ = 0.42, p < 0.001), MCI-AD (r_s_ = 0.43, p < 0.01), and sMCI (r_s_ = 0.52, p < 0.001), whereas CSF YKL-40 was not correlated with CSF NFL in the MCI-VaD group (r_s_ = −0.01). Correlations were sought using the Spearman rank order correlation test. Aβ: amyloid-β; AD: Alzheimer's disease; CSF: cerebrospinal fluid; MCI: mild cognitive impairment; NFL: neurofilament light chain; P-tau_181_: phosphorylated tau_181_; sMCI: stable MCI; T-tau: total tau; VaD: vascular dementia.

Baseline correlations between the CSF biomarkers are given in Table 3. In addition, correlations between neurogranin and the biomarkers Aβ_42_, t-tau, p-tau_181_, and NFL are given in Figure 2, and correlations between YKL-40 and these biomarkers are presented in Figure 3. Neurogranin and YKL-40 correlated positively with each other in the sMCI group (r_s_ = 0.45, p < 0.01), but not in the other groups. Neurogranin correlated positively and markedly with t-tau and p-tau_181_ in all study groups (Table 3 and Figure 2). Furthermore, neurogranin correlated positively with CSF Aβ_42_ level in the MCI-VaD and sMCI groups, but not in the MCI-AD group. Neurogranin was only correlated with NFL in the sMCI group (Table 3 and Figure 2).

**Table 3.** Baseline correlations between CSF levels of neurogranin, YKL-40, the core AD biomarkers, and NFL in 107 subjects with MCI who had not yet sought help for their cognitive decline.

	MCI-AD (n = 40)		MCI-VaD (n = 25)		sMCI (n = 42)	
	Neurogranin (ng/L)	YKL-40 (ng/mL)	Neurogranin (ng/L)	YKL-40 (ng/mL)	Neurogranin (ng/L)	YKL-40 (ng/mL)
Neurogranin (ng/L)		r_s_ = 0.24, p = 0.14		r_s_ = 0.30, p = 0.14		**r_s_** **=** **0.45, p** **<** **0.01**
YKL-40 (ng/mL)	r_s_ = 0.24, p = 0.14		r_s_ = 0.30, p = 0.14		**r_s_** **=** **0.45, p** **<** **0.01**	
Aβ_42_ (ng/L)	r_s_ = 0.00, p = 0.98	r_s_ = −0.20, p = 0.21	**r_s_** **=** **0.51, p** **=** **0.01**	r_s_ = 0.08, p = 0.72	**r_s_** **=** **0.43, p** **<** **0.01**	**r_s_** **=** **0.53, p** **<** **0.001**
t-tau (ng/L)	**r_s_** **=** **0.70, p** **<** **0.001**	**r_s_** **=** **0.44, p** **<** **0.01**	**r_s_** **=** **0.80, p** **<** **0.001**	r_s_ = 0.24, p = 0.26	**r_s_** **=** **0.78, p** **<** **0.001**	**r_s_** **=** **0.64, p** **<** **0.001**
p-tau_181_ (ng/L)	**r_s_** **=** **0.71, p** **<** **0.001**	r_s_ = 0.30, p = 0.06	**r_s_** **=** **0.80, p** **<** **0.001**	**r_s_** **=** **0.40, p** **=** **0.045**	**r_s_** **=** **0.77, p** **<** **0.001**	**r_s_** **=** **0.67, p** **<** **0.001**
NFL (ng/L)	r_s_ = −0.18, p = 0.27	**r_s_** **=** **0.43, p** **<** **0.01**	r_s_ = 0.09, p = 0.67	r_s_ = −0.01, p = 0.95	**r_s_** **=** **0.36, p** **=** **0.02**	**r_s_** **=** **0.52, p** **<** **0.001**

Correlations were calculated using the Spearman rank order correlation test. Rho values are presented as r_s_. Significant correlations are reported as bold text.

Aβ: amyloid-β; AD: Alzheimer's disease; CSF: cerebrospinal fluid; MCI: mild cognitive impairment; NFL: neurofilament light chain; P-tau_181_: phosphorylated tau_181_; sMCI: stable MCI; T-tau: total tau; VaD: vascular dementia.

YKL-40 correlated markedly with t-tau and p-tau_181_ levels in the sMCI group, whereas these correlations were weaker or absent in the MCI-AD and MCI-VaD groups (Table 3 and Figure 3). YKL-40 correlated positively with Aβ_42_ only in the sMCI group. YKL-40 correlated positively with NFL in the MCI-AD and sMCI groups (Table 3 and Figure 3).

We also investigated whether there were baseline correlations between neurogranin and YKL-40 and the neuropsychological test scores. However, we only found a positive correlation between neurogranin and immediate recall from the CAB in the MCI-VaD group (r_s_ = 0.40, p = 0.049). Otherwise, neurogranin or YKL-40 did not correlate with the neuropsychological test results in any study group (data not shown).

### ROC analyses

**Figure 4.**
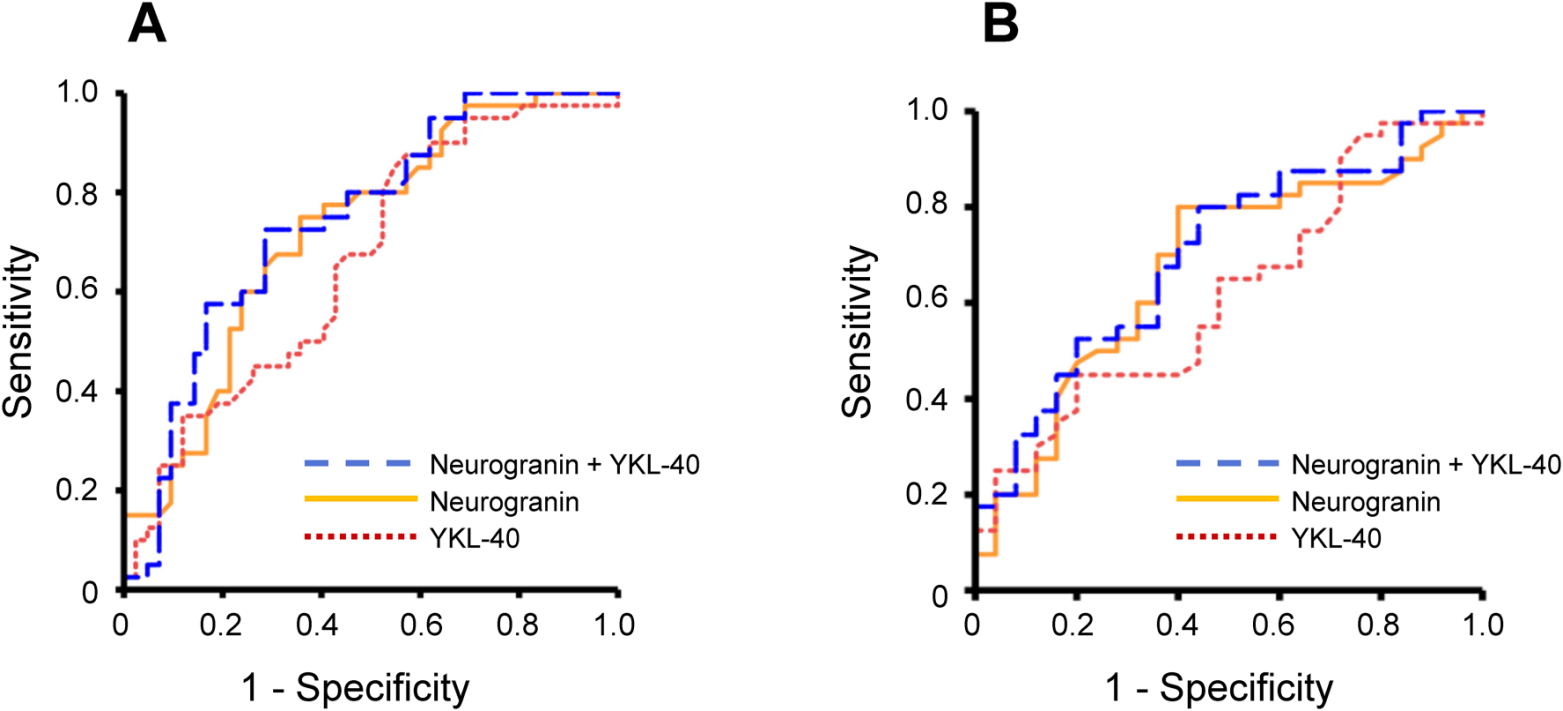
The separation of MCI-AD (n = 40) from sMCI (n = 42) and MCI-VaD (n = 25) using CSF levels of neurogranin and YKL-40. The results of ROC statistics are presented. (A) MCI-AD was significantly discriminated from sMCI by neurogranin (AUROC 0.721, 95% CI: 0.610–0.831, p < 0.001), YKL-40 (AUROC 0.661, 95% CI: 0.543–0.779, p = 0.01), and the combined use of neurogranin and YKL-40 (AUROC 0.743, 95% CI: 0.636–0.851, p < 0.001). (B) MCI-AD was significantly differentiated from MCI-VaD by neurogranin (AUROC 0.676, 95% CI: 0.541–0.811, p = 0.02) and the combined use of neurogranin and YKL-40 (AUROC 0.702, 95% CI: 0.573–0.831, p < 0.01), whereas MCI-AD was not significantly separated from MCI-VaD by YKL-40 used alone (AUROC 0.620, 95% CI: 0.481–0.758, p = 0.11). In the panels, orange solid line represents neurogranin; red dotted line represents YKL-40, and blue dashed line shows neurogranin combined with YKL-40. AD: Alzheimer's disease; AUROC: area under the receiver operating characteristics curve; CSF: cerebrospinal fluid; MCI: mild cognitive impairment.; ROC: receiver operating characteristics; sMCI: stable MCI; VaD: vascular dementia.

In ROC analyses, neurogranin in CSF had a moderate ability to separate MCI-AD from sMCI (AUROC 0.721, 95% CI: 0.610–0.831, p < 0.001; Figure 4A) and to distinguish MCI-AD from MCI-VaD (AUROC 0.676, 95% CI: 0.541–0.811, p = 0.02; Figure 4B). YKL-40 in CSF modestly discriminated MCI-AD from sMCI (AUROC 0.661, 95% CI: 0.543–0.779, p = 0.01; Figure 4A), whereas YKL-40 was not able to significantly separate MCI-AD from MCI-VaD (AUROC 0.620, 95% CI: 0.481–0.758, p = 0.11; Figure 4B). Finally, as both neurogranin and YKL-40 were higher in MCI-AD compared with sMCI, we investigated if the combined use of these biomarkers could increase the diagnostic accuracy. However, neurogranin and YKL-40 used in combination only marginally increased the capacity to distinguish MCI-AD from sMCI (AUROC 0.743, 95% CI: 0.636–0.851, p < 0.001; Figure 4A) or MCI-AD from MCI-VaD (AUROC 0.702, 95% CI: 0.573–0.831, p < 0.01; Figure 4B).

### Subanalyses

We performed subanalyses to determine whether neurogranin and YKL-40 were altered in subgroups of MCI-AD or MCI-VaD. In the MCI-AD group (n = 40), CSF neurogranin was similar in the 26 participants with MCI-AD not having any major cerebral vascular comorbidity (median: 216 ng/L, 25^th^–75^th^ percentiles: 177–239 ng/L) as that in the 14 participants with MCI-mixed dementia (229, 192–272 ng/L; p = 0.29). In the MCI-VaD group (n = 25), there was no difference in CSF neurogranin between the 18 participants with MCI-SVD (171, 152–218 ng/L) and the 7 subjects with MCI-cVaD (167, 147–244 ng/L; p = 0.74).

CSF YKL-40 did not differ significantly between MCI-AD without major cerebral vascular comorbidity (median: 158, 25^th^–75^th^ percentiles: 147–205 ng/mL) and MCI-mixed dementia (145, 123–241 ng/mL; p = 0.47), and there was no difference between MCI-SVD (151, 129–176 ng/mL) and MCI-cVaD (147, 105–173 ng/mL; p = 0.69).

## Discussion

In this single-center study, we recruited individuals who, after the medical examination, were found to have MCI. None of the subjects had any previous diagnosis of a cognitive disorder and none of the participants were under evaluation for cognitive decline when included in the study. Based on the baseline characteristics and the clinical progression at a 3-year follow-up, the participants received baseline diagnoses of MCI-AD, MCI-VaD, and sMCI. We found that CSF levels at baseline of neurogranin and YKL-40 were elevated in subjects with MCI-AD compared with those with sMCI. In addition, baseline CSF neurogranin was higher in MCI-AD than in MCI-VaD. CSF neurogranin, and to a lesser degree also CSF YKL-40, correlated positively with CSF levels of t-tau and p-tau_181_ in all study groups. Finally, in ROC analyses, neurogranin and YKL-40 used alone or in combination had a moderate diagnostic accuracy.

Our study population comprised elderly community-dwelling individuals with MCI who had not yet sought help at a memory clinic for their cognitive decline. As could be expected, the neuropsychological test results showed moderate decreases in cognition, and there were no between-group differences in neuropsychological test results except for SDMT, which was lower (worse) in MCI-VaD compared with sMCI. Finally, the levels of the core CSF AD biomarkers were, as expected, impaired in MCI-AD compared with the other study groups.

CSF levels of both neurogranin and YKL-40 were increased in the MCI-AD group compared with the sMCI group. Furthermore, the presence of concomitant cerebral vascular pathologies did not affect CSF levels of neurogranin and YKL-40 in MCI-AD as our subanalyses showed similar neurogranin and YKL-40 levels in MCI-AD without major cerebral vascular comorbidity as in MCI-mixed dementia (combined MCI-AD and MCI-VaD). Previously, higher CSF neurogranin levels have been found in patients with prodromal or manifest AD dementia compared with healthy controls^6–14^ or individuals under evaluation for other neurological disorders.^6,7,13,15,16^ Similarly, CSF YKL-40 level has been higher in AD dementia than in controls.^23,^^28–33^ Moreover, in memory clinic populations, CSF levels of neurogranin^11,14,15^ and YKL-40^32^ have mostly, but not always,^23^ been higher in MCI-AD patients compared with sMCI patients. Here, in individuals who had not yet sought help for their cognitive decline, MCI-AD was associated with higher CSF levels of neurogranin and YKL-40 compared with sMCI.

We found that CSF neurogranin level was higher in the MCI-AD group not only when compared with the sMCI group, but also when compared with the MCI-VaD group. Furthermore, in our subanalyses, CSF neurogranin level was similar in MCI-SVD and MCI-cVaD. In population-based cohorts, CSF neurogranin level was not associated with the risk of stroke.^53,54^ A few previous studies have evaluated CSF neurogranin levels in VaD patients.^21–24^ One study did not find any statistically significant difference in CSF neurogranin levels between AD patients (n = 64) and VaD patients (n = 18).^21^ However, in two studies, higher CSF neurogranin levels were observed in AD compared with cVaD.^23,24^ In another study, CSF neurogranin level was higher in AD dementia compared with patients with SVD.^22^ In summary, earlier studies have shown that CSF neurogranin can distinguish AD dementia from VaD, and we additionally demonstrate that CSF neurogranin level can separate MCI-AD from MCI-SVD and MCI-cVaD.

CSF YKL-40 level was higher in MCI-AD than in sMCI, but we found no difference between MCI-AD and MCI-VaD or between MCI-VaD and sMCI. In some accordance with these results, studies by Janelidze et al.^23^ and Llorens et al.^27^ showed similar CSF YKL-40 levels in AD and cVaD, whereas only the AD group had significantly higher CSF YKL-40 levels compared with controls.^23,27^ In a study by Olsson et al., CSF YKL-40 level was higher in MCI patients that later converted to SVD or cVaD compared with sMCI patients.^37^ Finally, one study showed similar CSF YKL-40 level in SVD as in idiopathic normal pressure hydrocephalus.^38^ Therefore, overall, there are few and somewhat conflicting data in terms of YKL-40 levels in VaD. Our results combined with the previous results could suggest that CSF YKL-40 is increased in MCI-VaD and manifest VaD, but that this increase is less marked than that in AD. Alternatively, there could be a specific VaD subgroup that has increased CSF YKL-40 levels. However, we found similar CSF YKL-40 levels in MCI-SVD and MCI-cVaD, which to some extent argue against the latter assumption.

In our ROC analyses, CSF neurogranin had a moderate ability to separate MCI-AD from sMCI (AUROC 0.721) and a slightly lower capacity to distinguish MCI-AD from MCI-VaD (AUROC 0.676). Even so, the diagnostic accuracy of CSF neurogranin was higher than that of CSF YKL-40 (MCI-AD vs. sMCI: AUROC 0.661; MCI-AD vs. MCI-VaD: not significant). Furthermore, the diagnostic accuracy of neurogranin and YKL-40 was moderate also when they were used in combination (MCI-AD vs. sMCI: AUROC 0.743). This is consistent with the findings that neurogranin and YKL-40 provide clearly lower diagnostic information compared with the core CSF AD biomarkers (Aβ_42_, Aβ_42_/Aβ_40_ ratio, t-tau, and p-tau_181_).^29,55^ However, CSF measurements of neurogranin and YKL-40 may still be valuable as they can provide information on the consequences of synaptic dysfunction and neuroinflammation. Earlier, higher CSF neurogranin level has been associated with more marked cognitive decline,^52^ lower brain cortical thickness,^56^ brain atrophy,^10^ and decreased glucose metabolism and higher hippocampal atrophy rates.^14^ Moreover, CSF YKL-40 levels have correlated negatively with brain cortical thickness in AD-vulnerable areas in Aβ_42_-positive subjects,^57^ and with brain grey matter volume in *APOE* ε4 carriers.^58^ Finally, in the future, measurements of neurogranin or YKL-40 could be useful to monitor treatment effects if medical therapies can be developed that target synaptic dysfunction and neuroinflammation in AD.

In the present study, CSF neurogranin level was markedly correlated with CSF levels of t-tau and p-tau_181_ in all study groups. In line with these findings, earlier studies have shown that CSF neurogranin levels correlate with both t-tau and p-tau in cognitively unimpaired subjects^8,15^ as well as in cognitively impaired individuals^6,8,9,15^ including AD patients.^6,8,9,13,15^ Although the mechanisms underlying these correlations are not fully clear, our findings combined with the previous results^6,8,9,13,15^ may suggest that synaptic dysfunction was linked to tau-related neurodegeneration. In contrast, but in accordance with previous results,^6,9^ the correlations between neurogranin and Aβ_42_ were not as strong and only seen in the MCI-VaD and sMCI groups. Possibly, amyloid pathology is necessary for the initiation of disease pathology, but to a lesser extent associated with on-going synapse loss in AD.

Compared with neurogranin, YKL-40 correlated weaker with CSF levels of t-tau and p-tau_181_. Moreover, there was a positive correlation between CSF YKL-40 level and CSF Aβ_42_ level only in the sMCI group. Previously, YKL-40 has been upregulated in inflammatory conditions,^26^ YKL-40 has been considered as a marker of neuroinflammation,^27^ YKL-40 expression has been abundant in astrocytes near Aβ plaques,^27^ and CSF YKL-40 levels have been positively correlated with CSF levels of t-tau and p-tau.^23,28,^^59–62^ However, in several studies, correlations between CSF levels of YKL-40 and Aβ_42_ have been absent or relatively weak in cognitively unimpaired individuals^23,60,61^ and in cognitively impaired individuals including AD.^23,28,59,61,62^ Therefore, the role of YKL-40 is not fully clear, but the results of recent studies suggest that YKL-40 is involved in early AD progression.^59–61^ Especially, YKL-40 may be a link between Aβ toxicity, astrogliosis and tau-driven neurodegeneration.^59–61^

We found a weak correlation between neurogranin and immediate recall from the CAB in the MCI-VaD group. Otherwise, neurogranin or YKL-40 did not correlate with the neuropsychological test results. The reason for the few correlations between neurogranin or YKL-40 and cognition in our study is unknown, but it could be of importance that in our community-based MCI population, the participants had a relatively modest decline in cognitive function.

Strengths of the present study include the well-defined study population and that the study was performed at a single center (Skaraborg Central Hospital). The study procedures were highly standardized and CSF levels of neurogranin and YKL-40 were determined at one occasion (September 2021). However, in each individual, CSF was only available at one timepoint. Therefore, we cannot determine whether longitudinal changes in neurogranin or YKL-40 levels were associated with the risk of progression to AD. Furthermore, the number of participants was limited, which could have reduced the statistical power. An additional study limitation is that the individuals in the MCI-AD and MCI-VaD groups were older than the individuals in the sMCI group. Moreover, all participants were of Caucasian origin, which may reduce the generalizability of our findings to other populations.

Another study limitation is that we did not have access to functional MRI (fMRI), which can evaluate the functional activity and connectivity of brain regions.^63^ Studies using fMRI have shown disruptions in functional connectivity networks, especially in regions associated with memory such as the hippocampus, which is an early target of synaptic damage in AD.^63,64^ Furthermore, we did not perform measurements of biomarkers in blood. In terms of neurogranin, plasma levels of neurogranin have not discriminated AD patients from controls.^12,65^ However, measurements of neurogranin in neuronally derived exosomes in plasma have shown more promise as these levels have been lower in AD patients compared with controls^66–69^ and in individuals with MCI-AD compared with stable MCI subjects.^67^ Plasma YKL-40 has also been assessed as an AD biomarker, and elevated levels have been found in patients with mild AD^62^ and early AD^70^ compared with controls. However, plasma YKL-40 showed low utility for predicting cognitive decline.^62^

In summary, in a single-center study comprising subjects with MCI who had not yet sought help for their cognitive decline, CSF levels of neurogranin and YKL-40 were elevated in MCI-AD compared with sMCI. Additionally, CSF neurogranin was higher in MCI-AD than in MCI-VaD. The correlation analyses suggested that synaptic dysfunction, and to some extent also neuroinflammation, was linked to tau-related neurodegeneration. In ROC analyses, the diagnostic accuracy of neurogranin and YKL-40 was moderate, but CSF measurements of neurogranin and YKL-40 may still be of value as they can provide information on the consequences of synaptic dysfunction and neuroinflammation. Especially, in the future, measurements of neurogranin or YKL-40 may be useful to monitor treatment effects if medical therapies can be developed that target synaptic dysfunction and neuroinflammation in AD.
